# P3HT Processing Study for In-Liquid EGOFET Biosensors: Effects of the Solvent and the Surface

**DOI:** 10.3390/s19204497

**Published:** 2019-10-17

**Authors:** Matteo Parmeggiani, Alessio Verna, Alberto Ballesio, Matteo Cocuzza, Erik Piatti, Vittorio Fra, Candido Fabrizio Pirri, Simone Luigi Marasso

**Affiliations:** 1 Department of Applied Science and Technology (DISAT), Politecnico di Torino, Corso Duca degli Abruzzi 24, 10129 Torino, Italy; alessio.verna@polito.it (A.V.); alberto.ballesio@polito.it (A.B.); matteo.cocuzza@infm.polito.it (M.C.); erik.piatti@polito.it (E.P.); vittorio.fra@polito.it (V.F.); fabrizio.pirri@polito.it (C.F.P.); 2 Istituto Italiano di Tecnologia, Center for Sustainable Future Technologies, Via Livorno 60, 10144 Torino, Italy; 3 Istituto dei Materiali per l’Elettronica ed il Magnetismo, IMEM-CNR, Parco Area delle Scienze 37/A, 43124 Parma, Italy

**Keywords:** biosensor, bioelectronics, EGOFET

## Abstract

In-liquid biosensing is the new frontier of health and environment monitoring. A growing number of analytes and biomarkers of interest correlated to different diseases have been found, and the miniaturized devices belonging to the class of biosensors represent an accurate and cost-effective solution to obtaining their recognition. In this study, we investigate the effect of the solvent and of the substrate modification on thin films of organic semiconductor Poly(3-hexylthiophene) (P3HT) in order to improve the stability and electrical properties of an Electrolyte Gated Organic Field Effect Transistor (EGOFET) biosensor. The studied surface is the relevant interface between the P3HT and the electrolyte acting as gate dielectric for in-liquid detection of an analyte. Atomic Force Microscopy (AFM) and X-ray Photoelectron Spectroscopy (XPS) characterizations were employed to study the effect of two solvents (toluene and 1,2-dichlorobenzene) and of a commercial adhesion promoter (Ti Prime) on the morphological structure and electronic properties of P3HT film. Combining the results from these surface characterizations with electrical measurements, we investigate the changes on the EGOFET performances and stability in deionized (DI) water with an Ag/AgCl gate electrode.

## 1. Introduction

The promise for precise and low-cost health monitoring [1] to obtain an early stage alert on critical diseases (i.e., heart attacks, tumors, diabetes, etc.), as well as the interest to detect drugs and contaminants with potential carcinogenic effects in food [2,3] and water [4,5], has pushed the investigation on an increasing amount of significative biomarkers and analytes and, in parallel, a search for cost-effective and accurate methods for their recognition. To match this goal, a highly efficient solution is represented by electrochemical and electric based biosensors, a class of devices that are easy to integrate with consumer electronics [6] already present in smartphones, cars, and domotic systems, processable on flexible substrates [7], to be applied in smart textiles [8], accurate and tailorable to detect different analytes [9,10]. In this view, a must for these kinds of devices is represented by the response stability, a requirement which is often in contrast with the used materials, especially when polymers are employed. On the other hand, the devices’ production can enormously take advantage of the use of polymers in terms of cost and processability. Hence, recent studies have been focused on processing and materials testing with the final aim to increase device performances and stability [11,12]. Electrolyte Gated Organic Field Effect Transistors (EGOFETs) are emerging as a promising technology for biosensing applications due to their known high sensibility, low-voltage operation, biocompatibility and low-cost fabrication [13].

Poly(3-hexylthiophene) (P3HT) is one of the most used semiconductive polymers for these kinds of devices due to its relatively high carrier mobility and easy processability [14]. Recent works have exploited P3HT as an organic semiconductor for EGOFET biosensors [15], but the main drawback still affecting these devices is their fast degradation when working in ambient conditions [16], also with respect to similar devices like Organic Electro Chemical Transistors (OECTs), which exhibit a better stability in-liquid [17] and a tailorable conductive behavior [18]. Previous studies report a correlation between the organic semiconductor behavior and the rearrangement induced by the surface [19,20], which is related to different factors, such as type of substrates, deposition methods, and thermal treatments. Moreover, for an EGOFET biosensor, the semiconductor surface represents the direct interface with the electrolyte solution, and hence it is mandatory to maintain its stability as much as possible to avoid signal drifting or device failure [21].

In this study we investigate the effect of solvents and adhesion promoters on the morphology and electronic structure of a P3HT thin film in order to improve the stability and electrical properties of an EGOFET biosensor. After the study of the surface properties and fabrication process optimization, we investigate the effects on the device by evaluating the performances and response stability in DI water with an Ag/AgCl gate electrode. 

## 2. Materials and Methods

### 2.1. Materials and Reagents

Poly(3-hexylthiophene-2,5-diyl) (P3HT) was purchased from Rieke Metals (M_w_ = 37 kDa, regioregularity > 96%, RMI001-EE), Ti prime adhesion promoter was purchased from MicroChemicals, all other chemicals were purchased from Sigma-Aldrich.

### 2.2. Device Fabrication

Standard clean room processes were employed to develop the EGOFET. A Ti adhesion layer (10 nm) and Au layer (100 nm) were e-beam evaporated on p-type (100) Si wafers finished with a 1000 nm SiO_2_ coating. Afterwards, source and drain interdigitated electrodes were photolithographically patterned and wet-etched (channel length L = 10 µm, channel width W = 9590 µm). After photolithography and etching, half of the samples were treated with the adhesion promoter. Ti prime was spin coated at 4000 rpm for 30 s and dried at 120 °C for 2 min on a hot plate. P3HT solutions were prepared with concentration of 2.5 mg/mL in toluene or in 1,2-dichlorobenzene (oDCB) and spin coated at 2000 rpm for 30 s. Finally, the devices were dried at 75 °C under vacuum for 1 h to completely remove any solvent residue. Samples for XPS and AFM analyses were fabricated following the same procedures but on clean Si/SiO_2_ substrates without patterned source and drain electrodes. The four different processes, namely No prime tol, Ti prime tol, No prime oDCB, and Ti prime oDCB are summarized in Table 1, the electrical characterizations have been performed on five devices for each process, the device with better performances was then used for the stability measurements. 

### 2.3. Characterizations

AFM characterizations have been performed in tapping mode with a Bruker Innova Atomic Force Microscope to monitor the quality of the P3HT film surface. 

XPS analysis has been performed using the X-ray source Al Kα 1486.6 eV, pass energy 187.85 eV for survey analysis, and 23.50 eV for peaks and valence band analysis.

Electrical characterizations have been performed in a probe station with a Keysight B2912A Source/Measure unit connected to the micromanipulators through triax cables. Resistances R have been extracted from I-V curves and sheet resistances of P3HT films (thickness t = 30 nm) have been estimated as $R_{s}=R\cdot \frac{W}{L}$. The results have been validated with sheet resistance measurements carried out exploiting the 4 probes method on 5 other different samples for each process. Then the devices were characterized in EGOFET configuration using DI water as gate electrolyte and an Ag/AgCl leak-free reference electrode as gate electrode. A PDMS well with volume of 60 μL was placed on top of the interdigitated electrodes and filled with DI water, the gate electrode was placed inside the well and contacted from top. Output and transfer characteristics were measured at different gate and drain voltages on each device, with a scan rate of 40 mV/s. In order to investigate device stability, on one device for each process the transfer curve has been measured multiple times (100 repetitions) at a scan rate of 40 mV/s. Transfer curves have also been measured at a scan rate = 400 mV/s in order to investigate the hysteresis and switching speed of the devices. The gate leakage current has been continuously monitored during all measurements

## 3. Results and Discussion

### 3.1. Tapping Mode AFM

**Figure 1:**
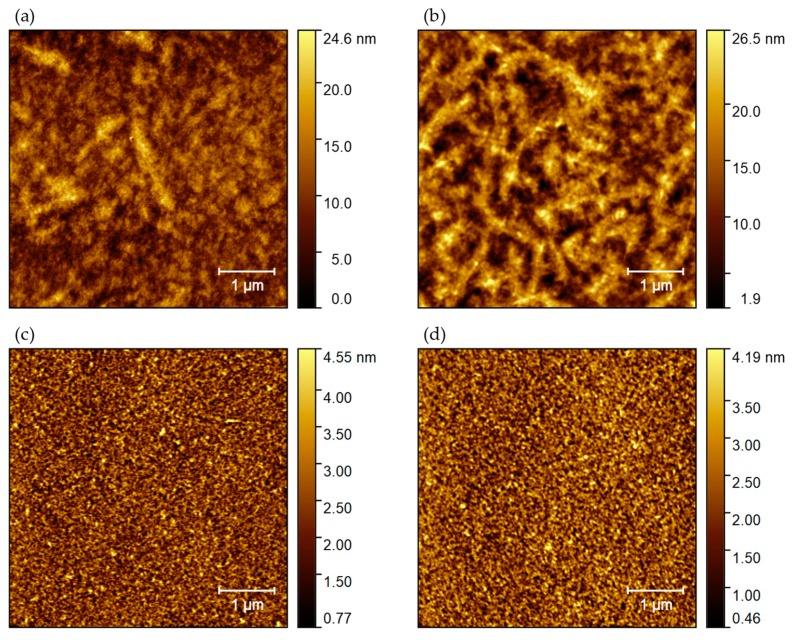
AFM topography maps acquired in tapping mode of P3HT deposited on Si/SiO_2_ substrate (**a**) from toluene solution without adhesion promoter, (**b**) from toluene solution with Ti prime, (**c**) from oDCB solution without adhesion promoter, and (**d**) from oDCB solution with Ti prime.

AFM analysis has been carried out on four different samples for each process (toluene or oDCB based P3HT solutions, with or without Ti prime). Figure 1a,b respectively show 5 μm × 5 μm topography maps of P3HT deposited using toluene solution on clean SiO_2_ substrate and on SiO_2_ treated with adhesion promoter. The presence of Ti prime increases the RMS surface roughness from 1.92±0.65 nm given by the No prime Tol process to 4.10±0.90 nm. In both cases the surface is strongly disordered. In contrast, films obtained using oDCB as solvent (Figure 1c,d) present a flatter surface topography with an RMS roughness of 0.78±0.08 nm for the No prime oDCB process and 0.74±0.18 nm for the Ti prime oDCB process, and organization in nanocrystalline domains.

### 3.2. XPS Characterization

XPS characterization has been performed on three samples for each set of devices in order to study the influence of different fabrication processes on the electronic properties of P3HT thin films. C1s, S2p, and valence band region high resolution spectra have been investigated. The energy scale was aligned fixing the C1s peak at 284.80 eV, all the peaks were fitted with mixed Gauss–Lorentzian lineshapes (90% Gaussian) and Shirley background.

**Figure 2:**
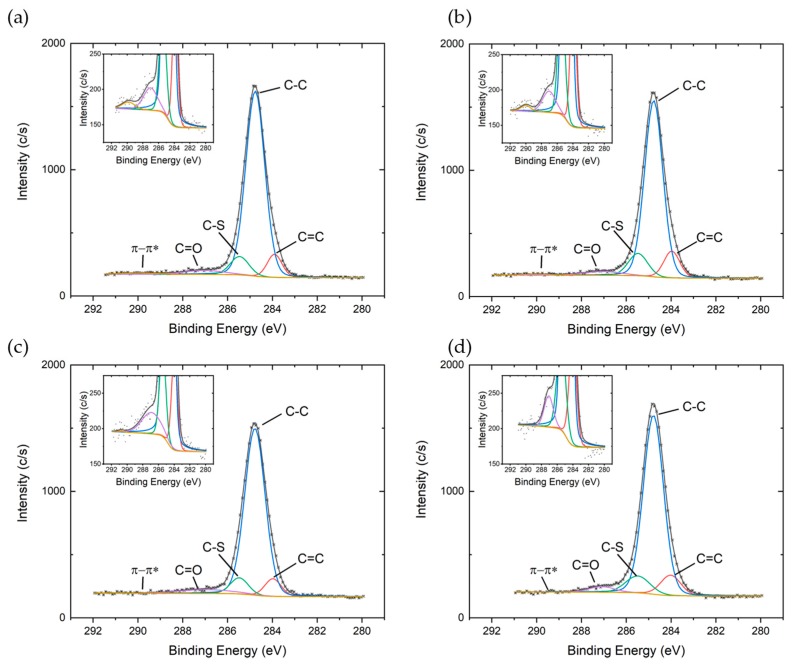
High resolution C1s spectra of P3HT deposited on Si/SiO_2_ substrate (**a**) from toluene solution without adhesion promoter, (**b**) from toluene solution with Ti prime, (**c**) from oDCB solution without adhesion promoter, and (**d**) from oDCB solution with Ti prime. The insets show a zoom on the π−π* shake-up satellite region.

C1s peaks (Figure 2) were deconvoluted with five components: the main component around 284.80 eV accounting for C–C bond atoms in in the alkyl side chains, two components around 284.00 eV and 285.50 eV accounting for C=C and C–S bonds in the thiophene ring respectively [22,23], a fourth component at around 2.5 eV higher binding energy with respect to the main one, related to carbonyl group [24] most probably formed due to incorporation of oxygen impurities during fabrication process, and a fifth component around 289.90 eV attributed to $\pi -\pi^{*}$ transitions.

The intensity of $\pi -\pi^{*}$ shake-up satellites can be used to qualitatively understand the degree of delocalization of π orbitals along the polymer backbones and inside the polymeric film; stronger satellite peaks are due to a less efficient screening of the core hole formed during the photoionization process and are associated with a lower degree of delocalization of π orbitals [25]. When using toluene as solvent (Figure 2a,b) the area percentage associated with $\pi -\pi^{*}$ satellites decreases from 0.60%±0.20% for the No prime tol process to 0.40%±0.15% for the Ti prime tol process, suggesting that Ti Prime may slightly improve the π stacking of polymer chains and reduce the conformational disorder along polymer backbones. When using oDCB as solvent, the intensity of the shake-up satellite is systematically lower than the background noise, indicating better delocalization of the molecular orbitals.

**Figure 3:**
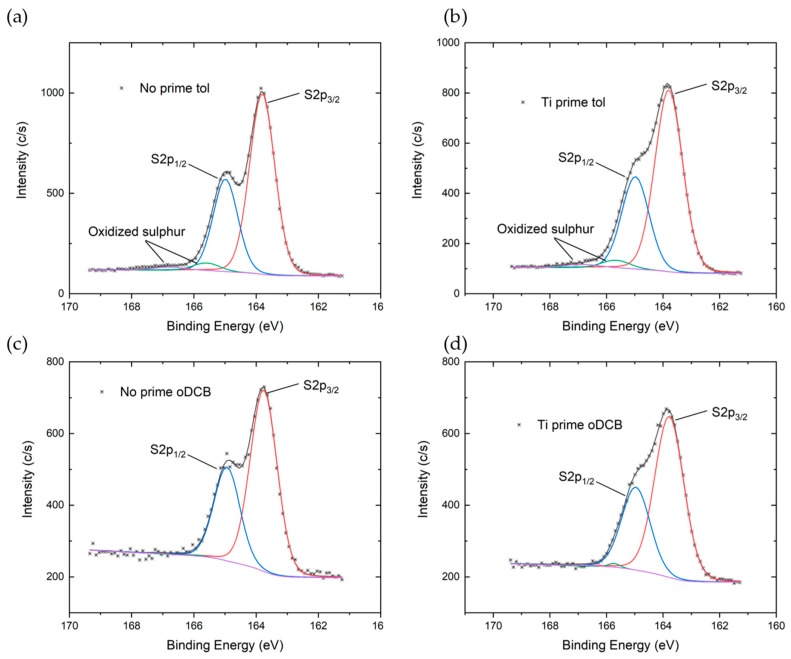
High resolution S2p spectra of P3HT deposited on Si/SiO_2_ substrate (**a**) from toluene solution without adhesion promoter, (**b**) from toluene solution with Ti prime, (**c**) from oDCB solution without adhesion promoter, and (**d**) from oDCB solution with Ti prime.

The S2p core-line (Figure 3) spectra have been fitted with four components. The first two relate to the spin orbit doublet of sulfur bonded only to carbon in the thiophene rings, with S2p_3/2_ centered around 163.80 eV, peak separation of 1.18 eV, and area ratio of ½. The other two components were used to fit a second doublet at higher binding energy (1.8–1.9 eV) related to sulfur bonded to more electronegative atoms, indicating sulfur oxidation in the thiophene ring. When using toluene as solvent the area percentage occupied by the second doublet decreases from 5.4%±1.9% for the No prime tol process to 4.3%±1.8% for the Ti prime tol process. When using oDCB, the same trend is obtained, with 4.6%±1.0% for No prime oDCB and 2.7%±0.6% for the Ti prime oDCB process. These results suggest that the use of oDCB as a solvent and Ti prime as an adhesion promoter may reduce sulfur oxidation during the fabrication process and thus polymer degradation. 

**Figure 4:**
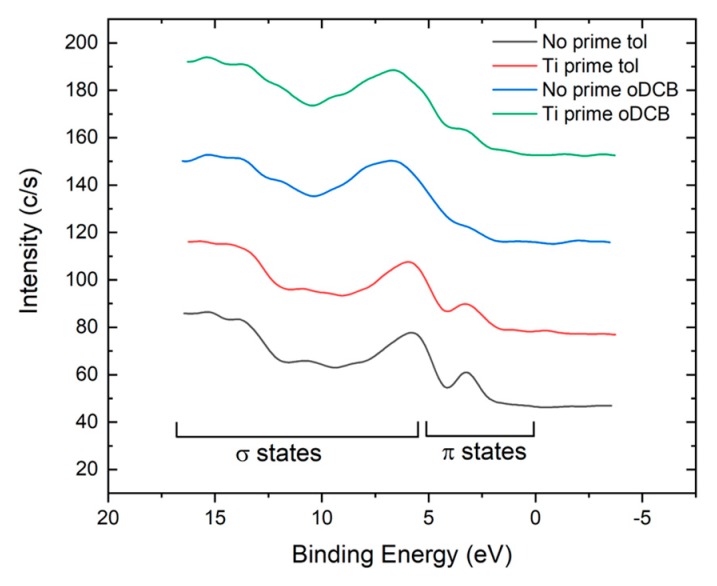
Density of states in the valence band region measured for the four different films.

The density of states in the valence band region has been investigated via XPS spectroscopy. Figure 4 shows the typical spectra obtained for the different processes. Samples fabricated with the same process did not show appreciable differences in the valence band region. Contributions at a high binding energy (B.E. > 5 eV) are due to binding σ orbitals from the alkyl chains, while between 0 and 5 eV there are only contributions coming from π orbitals of the conjugated backbones. The peak at 3.4 eV in the spectrum of P3HT deposited from the toluene solution without an adhesion promoter (black curve in Figure 4) can be attributed to localized states. A slight broadening is seen in the spectra corresponding to the Ti prime tol process (red curve in Figure 4). The broadening is more evident for P3HT deposited from oDCB solution and may be related to a higher degree of delocalization of the molecular orbital, in agreement with the disappearance of the shake-up satellites in C1s peaks. This indicates a higher degree of π–π conjugation of different polymer backbones, which is expected to improve the electrical properties of the material [24].

### 3.3. Electrical Characterization

**Figure 5:**
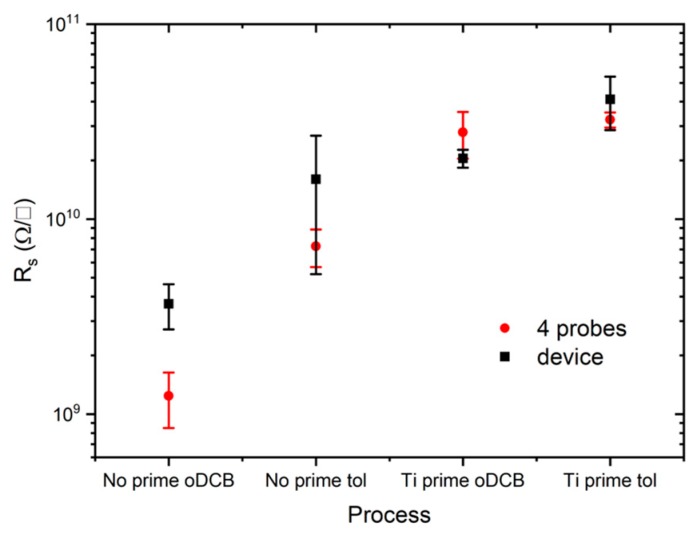
Sheet resistances extrapolated from a set of five devices for each process (black symbols) and measured with the four probes method on the other five samples for each process (red symbols). Devices fabricated starting from oDCB solution generally show higher conductivity with respect to those fabricated with toluene. Ti prime improves reproducibility but reduces the conductivity independently on the solvent.

Sheet resistance measurements (Figure 5) show that films obtained using oDCB as solvent are more conductive than the corresponding films obtained from toluene. This result supports the hypothesis of a better delocalization of states coming from the XPS spectra analysis when oDCB is used. In contrast to XPS analysis however the presence of the adhesion promoter lowers samples’ conductivity. When Ti prime is used, sheet resistances increase of one order of magnitude for films deposited from oDCB solution and double for films deposited from toluene solution. Samples treated with Ti prime show lower standard deviation independently on the solvent, suggesting that the presence of the adhesion promoter can improve the device-to-device reproducibility.

**Figure 6:**
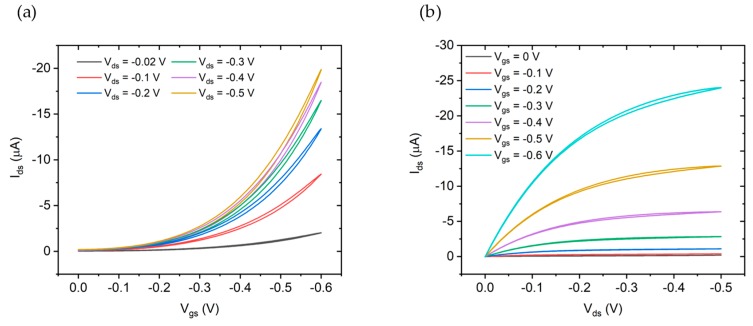
(**a**) Transfer characteristics and (**b**) output characteristics of the Electrolyte Gated Organic Field Effect Transistor (EGOFET) fabricated with no adhesion promoter using P3HT dissolved in oDCB. DI water has been used as a gate electrolyte and an Ag/AgCl leak free reference electrode as a gate electrode.

Transfer characteristics (I_ds_ vs. V_gs_) and output characteristics (I_ds_ vs. V_ds_) of a device fabricated without an adhesion promoter using oDCB as solvent are shown in Figure 6a,b respectively. Transfer and output characteristics have been measured on a set of five devices for each different process using DI water as a gate electrolyte and a leak free Ag/AgCl reference electrode as a gate electrode.

First of all, threshold voltages $V_{th}$ have been extrapolated from the linear fit of $\sqrt{I_{ds}}\;vs\;V_{gs}$ in the saturation region, where the drain current is given by Equation (1):

(1) $$I_{ds}=\frac{1}{2}\mu C_{g}\frac{W}{L}{\left(V_{gs}-V_{th}\right)}^{2}$$

with $C_{g}$ = 3 μF/cm^2^ as the gate capacitance [4].

**Figure 7:**
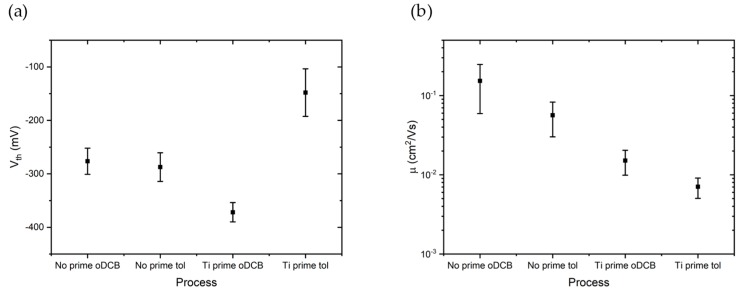
(**a**) Threshold voltage extrapolated from transfer characteristics of five devices for each process. (**b**) Field effect mobility obtained from output characteristics in linear regime of five devices for each process.

The mean $V_{th}$ obtained from five devices are shown in Figure 7a. Afterwards, the field-effect mobility μ of charge carriers in the P3HT deposited from toluene and oDCB, with and without Ti prime, has been extracted from the output characteristics in the linear region, where $\left|V_{gs}\right|>\left|V_{th}\right|,\;\left|V_{ds}\right|<\left|V_{gs}\right|-\left|V_{th}\right|$ and:

(2) $$I_{ds}=\mu C_{g}\frac{W}{L}\left(V_{gs}-V_{th}\right)V_{ds}$$

For each device, a linear fit of output curves measured at gate voltages higher (in absolute value) than the threshold voltage obtained from the corresponding transcharacteristics have been performed. μ has been calculated from the slope of the linear fit. Figure 7b shows the average mobilities obtained from output characteristics of five devices for each process. Devices fabricated without Ti prime show higher mobilities ($\mu =1.53\times 10^{-1}\frac{{\mathrm{cm}}^{2}}{Vs}$ for the No prime oDCB process and $5.6\times 10^{-2}\frac{{\mathrm{cm}}^{2}}{Vs}$ for the No prime tol process) with respect to devices fabricated with the adhesion promoter ($\mu =1.5\times 10^{-2}\frac{{\mathrm{cm}}^{2}}{Vs}$ for the Ti prime oDCB process and $7\times 10^{-3}\frac{{\mathrm{cm}}^{2}}{Vs}$ for the Ti prime tol process). Ti prime processes, on the other hand, have better device-to-device reproducibility.

Repeated measurements of transfer characteristics have been performed on one device for each process, at fixed $V_{ds}=-500\;\mathrm{mV}$, sweeping the gate voltage from 0 V to −600 mV and back. The cycle was repeated 100 times. Each of the 100 repeated measurements was acquired with one point every 10 mV and 250 ms of delay between subsequent points, resulting in a scan rate of 40 mV/s. Each single transfer curve took 30 s to be measured from 0 V to −0.6 V and back, and the following cycle started after 250 ms. Each stability test lasted 50 min in total. The transfer characteristics have been used for the stability analysis, monitoring the behavior of the threshold voltage $V_{th}$ and the transconductance $g_{m}$. The stability of these two parameters is of paramount importance in order to obtain reliable results during sensing experiments. Drift over time of these parameters should be taken into account when analyzing sensing results. In order to monitor transconductances variations, the maximum transconductance $g_{m,max}$ has been chosen as figure of merit and has been calculated as:

(3) $$g_{m,max}=\max\left(\frac{\partial I_{ds}}{\partial V_{gs}}\right)$$

**Figure 8:**
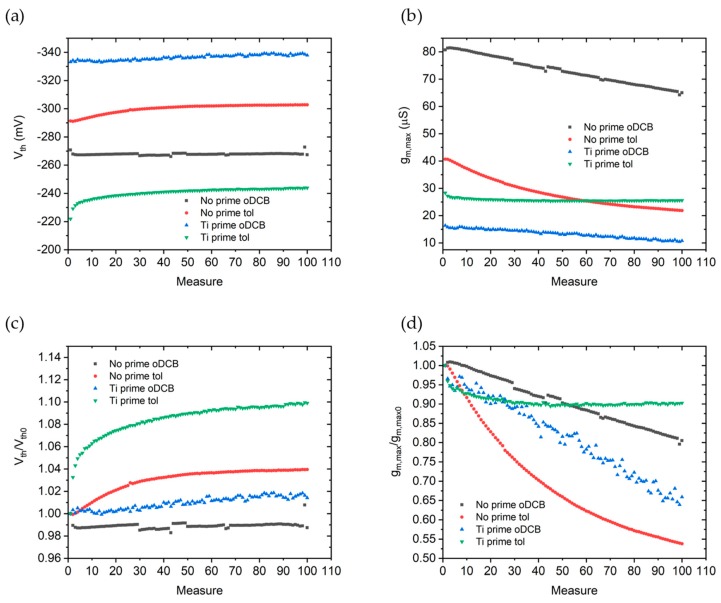
Threshold voltages (**a**) and maximum transconductances (**b**) extracted from 100 repeated $I_{ds}\;vs\;V_{gs}$ curves, measured at a scan rate = 40 mV/s. (**c**) Threshold voltages and (**d**) maximum transconductances normalized with respect to the first measurement.

In Figure 8a,b are shown the behaviors of threshold voltages and transconductances during repeated measurements. The same quantities normalized with respect to their initial value are reported respectively in Figure 8c,d.

Threshold voltages obtained for No prime oDCB and Ti prime oDCB are stable within 2% of their initial $V_{th}$, while No prime tol and Ti prime tol samples rapidly increase by 3% and 8% during the first 20 measurements, and then stabilize within 4% and 10% respectively.

The degradation of device performances when using oDCB can be primarily ascribed to a reduction of transconductance. As can be seen in Figure 8b,d, a linear decrease of $g_{m,max}$ is present both for No prime oDCB and Ti prime oDCB. When using toluene as a solvent instead, the presence of Ti prime enhances the transconductance stability.

**Figure 9:**
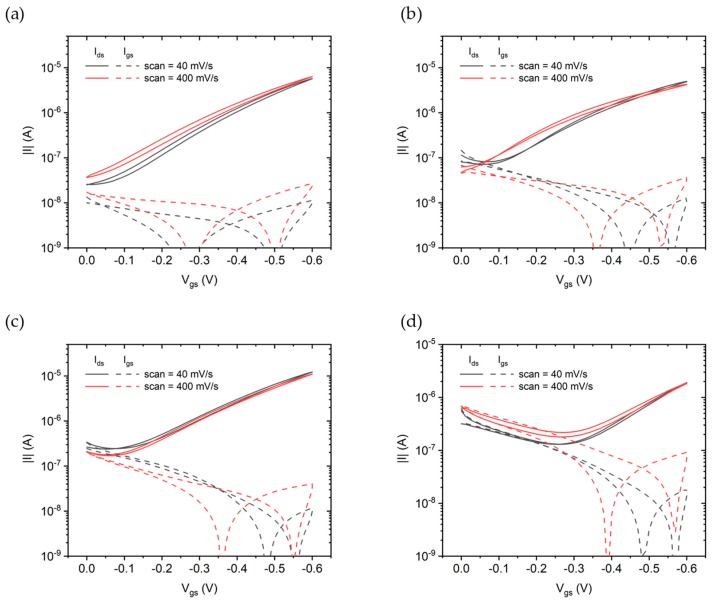
Transfer characteristics measured at scan rates of 40 mV/s (black curves) and 400 mV/s (red curves). Solid lines correspond to drain current while dashed lines are the gate leakage current. (**a**) No prime tol, (**b**) Ti prime tol, (**c**) No prime oDCB, and (**d**) Ti prime oDCB.

Transfer curves measured at different scan rates are shown in Figure 9. Independent of the scan speed, a small hysteresis is present on the drain currents, most likely due to trap filling at the semiconductor/electrolyte interface [26]. This indicates that the devices are operating in field effect mode, with negligible electrochemical doping. The gate leakage current is between two and three orders of magnitude lower than the drain current above threshold for almost all devices shown in Figure 9, with only the Ti prime oDCB (Figure 9d) showing a leakage current comparable to the drain one. The No prime tol sample exhibits the lower gate leakage and thus the higher I_on_/I_off_ ratio.

## 4. Conclusions

In conclusion, AFM and XPS results suggest that the use of oDCB improves the polymer crystallization, resulting in a film with a smoother surface and a higher degree of delocalization of electronic states at the HOMO level (valence band). Sheet resistance measurements quantitatively assess the improvement in conductivity due to the choice of the solvent and suggest that an adhesion-promoter treatment improves conductivity reproducibility. Finally, EGOFET’s characterizations demonstrate that a device fabricated exploiting oDCB as solvent with no adhesion promoter provide high response stability as shown by the repeated measurements, while, when using toluene, the adhesion promoter reduces the transconductance degradation. oDCB also seems to slightly prevent sulfur oxidation in the thiophene ring, reducing the fast degradation obtained for the EGOFET fabricated with toluene solution. Future investigations will be focused on testing the optimized EGOFETs in liquid medium of interest to detect biomarkers and analytes in human body (cells medium, plasma and blood) and in food (milk, honey, and eggs).

## Figures and Tables

**Table 1:** Devices were fabricated following four different processes. No prime tol and no prime oDCB are processes without adhesion promoter, using as solvent toluene or oDCB respectively. During Ti prime tol and Ti prime oDCB processes the samples were treated with adhesion promoter (Ti prime) before spin coating of poly(3-hexylthiophene) (P3HT) dissolved respectively in toluene or in 1,2-dichlorobenzene (oDCB).

Process	Solvent	Adhesion Promoter
No prime tol	Tol	✕
Ti prime tol	Tol	✓
No prime oDCB	oDCB	✕
Ti prime oDCB	oDCB	✓
